# A Rare Case of Multifocal Ileal and Mesenteric Gastrointestinal Stromal Tumors With Spontaneous Rupture

**DOI:** 10.7759/cureus.107748

**Published:** 2026-04-26

**Authors:** Aishvarya Vijayasekar, Gayathri Kuppusamy, Venkata Sai, Pavithra V, Ramana Sripathi

**Affiliations:** 1 Department of Radiodiagnosis, Sri Ramachandra Institute of Higher Education and Research, Chennai, IND; 2 Department of Pathology, Sri Ramachandra Institute of Higher Education and Research, Chennai, IND

**Keywords:** computed tomography, gastrointestinal stromal tumor, gist, peritoneal dissemination, pneumoperitoneum, small-bowel tumor, spontaneous rupture

## Abstract

Gastrointestinal stromal tumors (GISTs) most commonly present with gastrointestinal bleeding or as an abdominal mass, whereas spontaneous rupture with pneumoperitoneum is distinctly uncommon and portends an adverse prognosis. A 51-year-old man presented with acute hematemesis and melena of two days’ duration. Contrast-enhanced computed tomography of the abdomen demonstrated a large, lobulated, heterogeneously enhancing ileal mass with central necrosis, intralesional gas, direct communication with adjacent small-bowel loops, and free intraperitoneal air consistent with tumor rupture and pneumoperitoneum. Multiple additional mesenteric and pelvic masses with smooth peritoneal thickening and mild ascites suggested peritoneal dissemination. Emergency laparotomy confirmed tumor rupture with feculent peritonitis and widespread peritoneal seeding. Histopathology established a high-grade, multifocal GIST (pT4(m)N0) with spindle-to-epithelioid morphology, increased mitotic activity, necrosis, strong CD117 and DOG1 positivity, and an elevated Ki-67 labeling index. This case highlights the characteristic radiologic features of ruptured GIST and demonstrates close radiologic-pathologic concordance in an aggressive presentation.

## Introduction

Gastrointestinal stromal tumors (GISTs) are the most common mesenchymal neoplasms of the gastrointestinal (GI) tract, arising from the interstitial cells of Cajal. They account for less than 1% of GI malignancies, with an estimated annual incidence of 10-15 per million population. Although the stomach is the most frequent site, small-bowel GISTs constitute approximately one-quarter of cases and are associated with more aggressive biological behavior, larger size at presentation, higher mitotic rates, and a higher propensity for metastasis and an overall poorer prognosis [1,2].

Contrast-enhanced computed tomography (CECT) remains the primary imaging modality for diagnosis and staging, typically demonstrating well-defined, hypervascular, exophytic masses with varying degrees of necrosis or hemorrhage [1,3]. Spontaneous tumor rupture is uncommon, occurring at a rate of 5%-12% [4]. It is, however, clinically significant, as it is associated with peritoneal dissemination and is considered an independent adverse prognostic factor that upgrades the tumor to high-risk status irrespective of other parameters [5]. Early radiologic recognition is therefore critical in guiding urgent management.

## Case presentation

**Figure 1:**
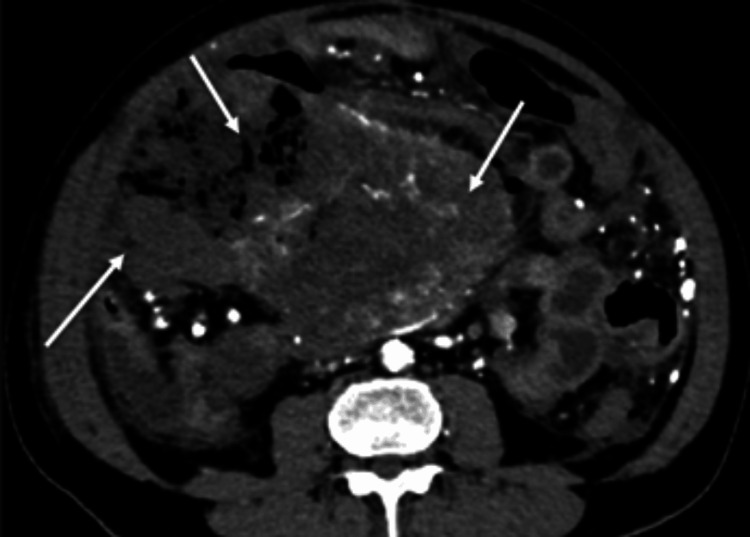
Axial CECT image in arterial phase showing a large lobulated heterogeneously hyperenhancing mass with central necrosis in the right lower quadrant (white arrows) CECT: contrast-enhanced computed tomography

A CECT of the abdomen was performed for further evaluation, which demonstrated findings suggestive of a complicated small-bowel neoplasm with perforation, prompting emergency surgical intervention. CECT demonstrated a large, predominantly exophytic, lobulated mass lesion, which measured approximately 11.0 x 16.3 x 18.5 cm (anteroposterior × transverse × craniocaudal) arising from the distal ileum in the right lower quadrant. The lesion showed heterogeneous arterial-phase hyperenhancement of viable tumor components with extensive central nonenhancing areas corresponding to necrosis (Figure 1), a pattern characteristic of large GISTs [1,3]. Arterial supply from branches of the superior mesenteric artery was identified (not shown in the figure), in keeping with the hypervascular nature of GIST [6].

**Figure 2:**
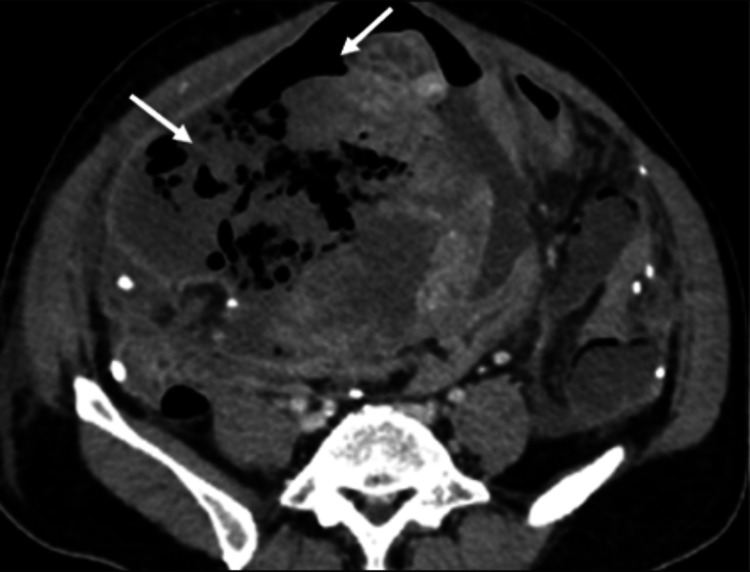
Axial CECT image in venous phase demonstrating exophytic mass with nondependent intratumoral air foci (white arrows) (Torricelli-Bernoulli sign) CECT: contrast-enhanced computed tomography

**Figure 3:**
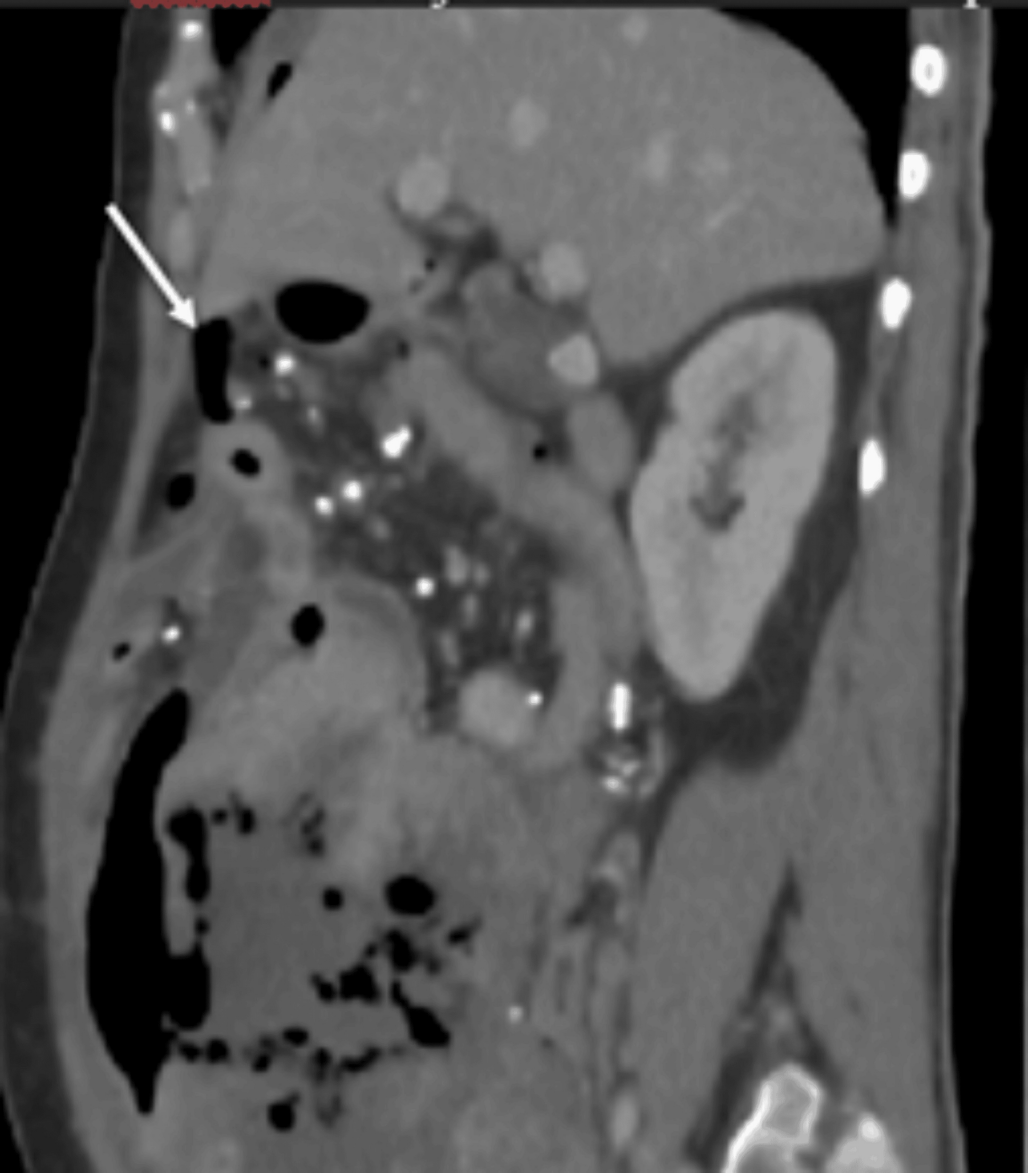
Sagittal CECT image in venous phase demonstrating pneumoperitoneum with air tracking from the tumor into the peritoneal cavity (white arrow) CECT: contrast-enhanced computed tomography

**Figure 4:**
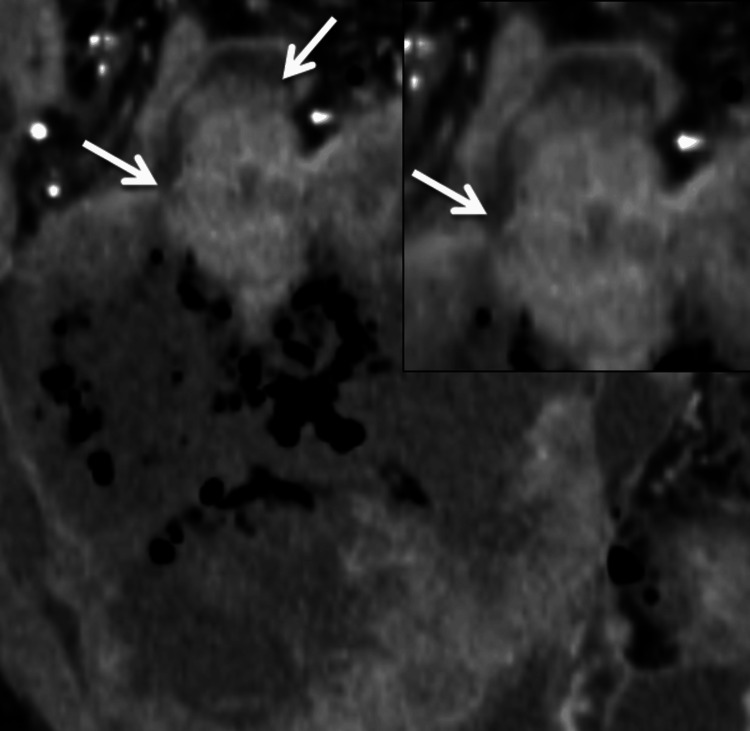
Coronal CECT image in venous phase demonstrating communication between the exophytic mass and adjacent small-bowel loops (white arrows) CECT: contrast-enhanced computed tomography

Multiple foci of intralesional gas were noted within the necrotic component (Figure 2). Importantly, linear air was seen tracking from the tumor into the peritoneal cavity through a focal defect, resulting in pneumoperitoneum (Figure 3). Direct communication between the mass and adjacent small-bowel loops was evident (Figure 4), indicating tumor-bowel fistulization and rupture. The presence of intratumoral gas in a long, untreated (approximately five years without therapy) lesion, particularly when associated with free intraperitoneal air, is highly suggestive of tumor rupture rather than simple necrosis [2]. The Torricelli-Bernoulli sign refers to the presence of nondependent intratumoral gas, resulting from pressure gradients that allow air to enter necrotic tumor cavities and indicate communication with the bowel lumen [7].

The lesion exerted mass effect on adjacent structures, displacing the ascending colon and indenting the urinary bladder, with loss of the fat plane with the urinary bladder, consistent with an expansile growth pattern and possibility of bladder infiltration.

**Figure 5:**
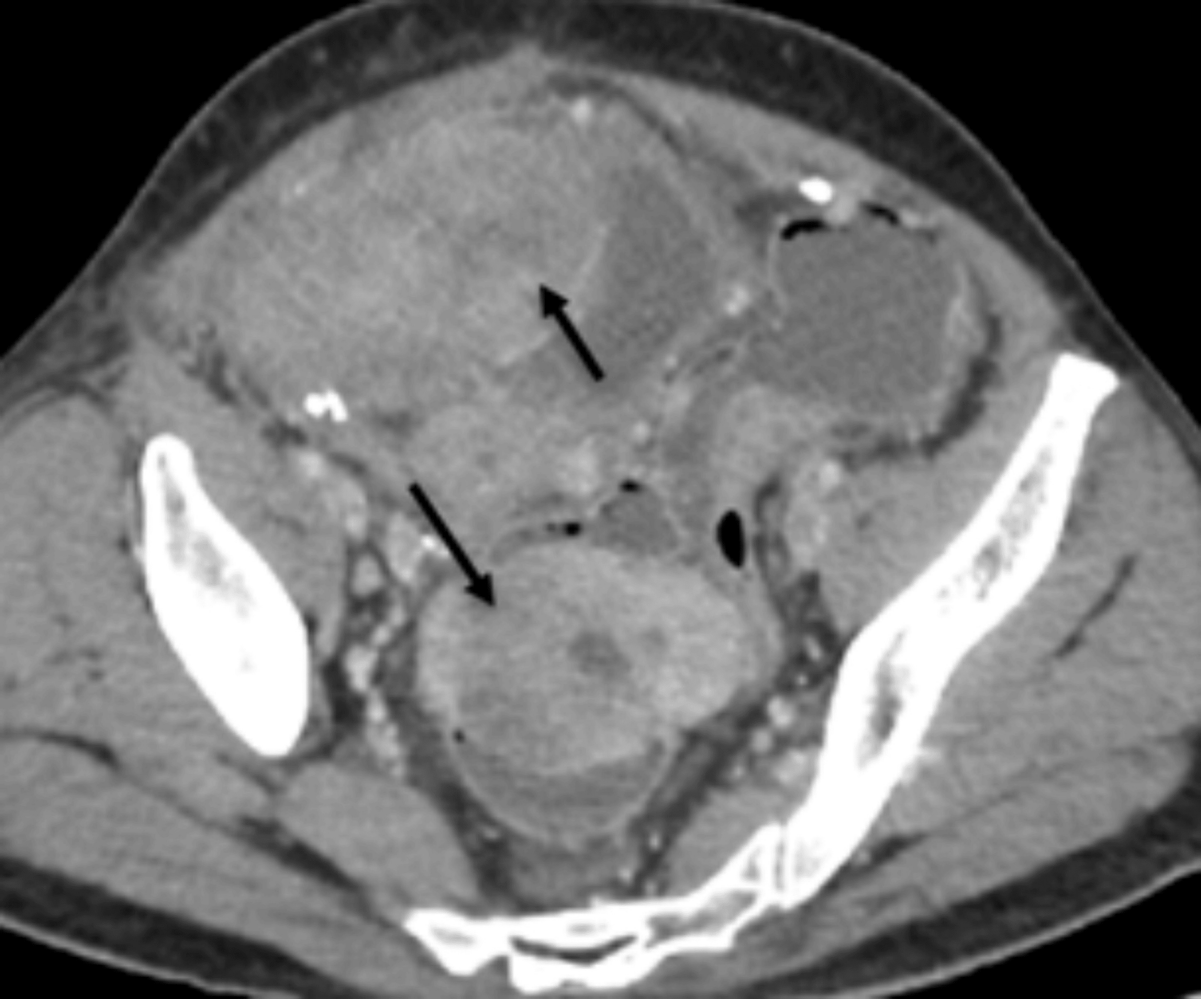
Axial CECT image in venous phase showing lobulated heterogeneously enhancing masses in the mesentery and pelvis (black arrows) CECT: contrast-enhanced computed tomography

Multiple additional lobulated, heterogeneously enhancing masses were identified within the mesentery and pelvis (Figure 5). Associated smooth peritoneal thickening and mild ascites were present, raising suspicion for peritoneal dissemination or multifocal primary tumors. No significant lymphadenopathy was identified, a finding that supports the diagnosis of GIST over other small-bowel malignancies [1,8].

**Figure 6:**
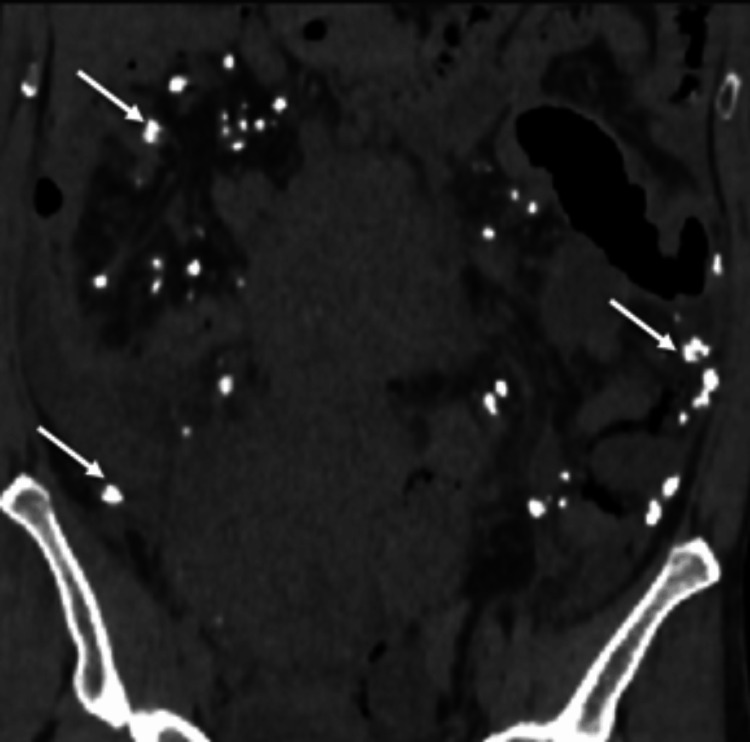
Coronal plain CT image showing multiple calcified mesenteric deposits (white arrows) CT: computed tomography

In addition, several completely calcified mesenteric deposits were noted, possibly related to treated metastatic disease (Figure 6).

These findings favored a ruptured multifocal GIST with peritoneal dissemination on a background of prior treated disease.

Intraoperative findings

Emergency laparotomy revealed a large ruptured tumor arising from the ileum approximately 20 cm proximal to the ileocecal junction. Approximately 500-600 mL of feculent and purulent fluid was present within the peritoneal cavity. Numerous tumor deposits were identified over the peritoneum, mesentery, and liver surface, along with dense interbowel adhesions. The tumor was found to infiltrate the urinary bladder, confirming the preoperative suspicion, necessitating partial cystectomy and repair. Segmental ileal resection with removal of tumor deposits and creation of an end ileostomy was performed.

Histopathology

**Figure 7:**
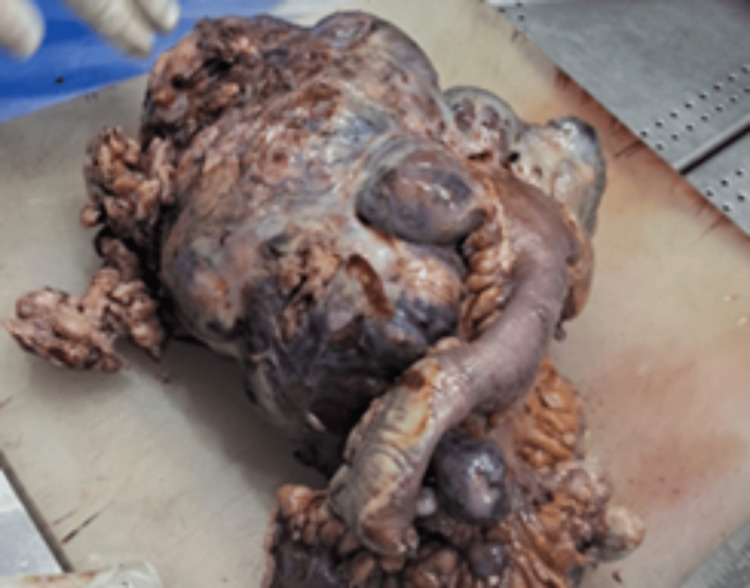
Gross specimen showing the resected ileal tumor with black necrotic areas along with ileum

**Figure 8:**
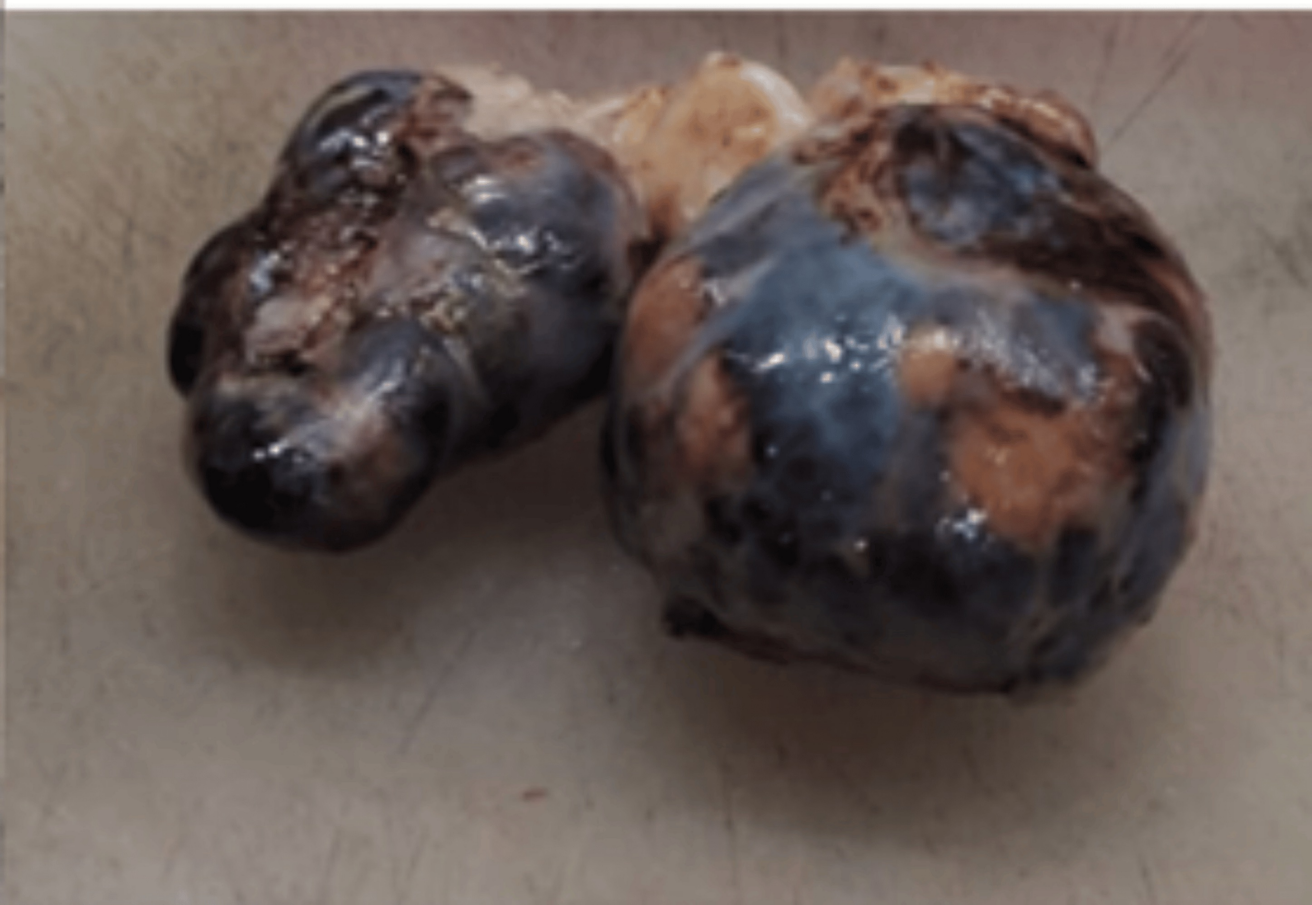
Gross specimen showing the resected mesenteric tumor deposits with black necrotic areas

Gross examination revealed a mesenteric-based mass measuring 16 × 14 × 8 cm with a large rupture defect. The cut surface was variegated with areas of necrosis and hemorrhage (Figures 7, 8), and multiple nodules were identified within the mesentery.

**Figure 9:**
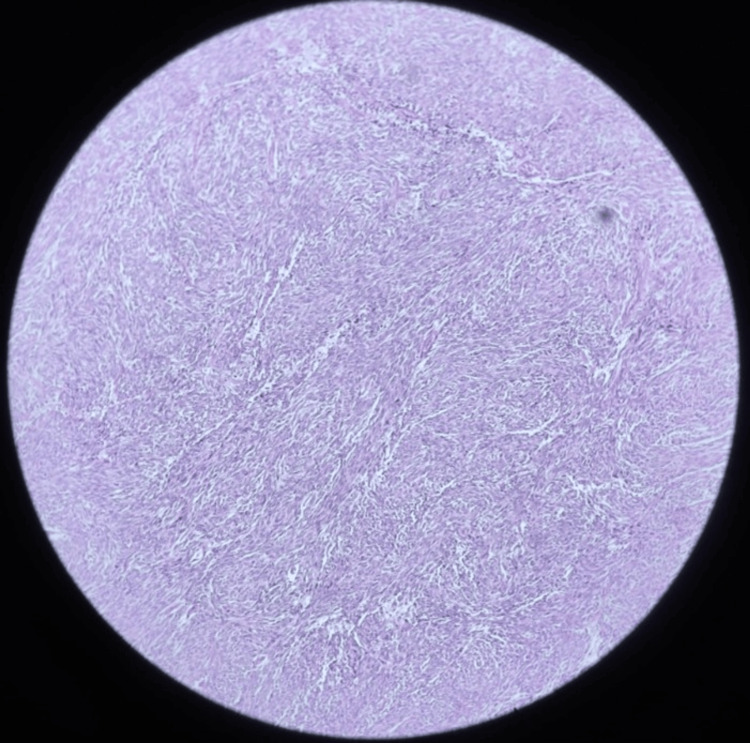
Microscopy (H&E, 10× power) showing spindle to epithelioid cells arranged in fascicles H&E: hematoxylin and eosin

**Figure 10:**
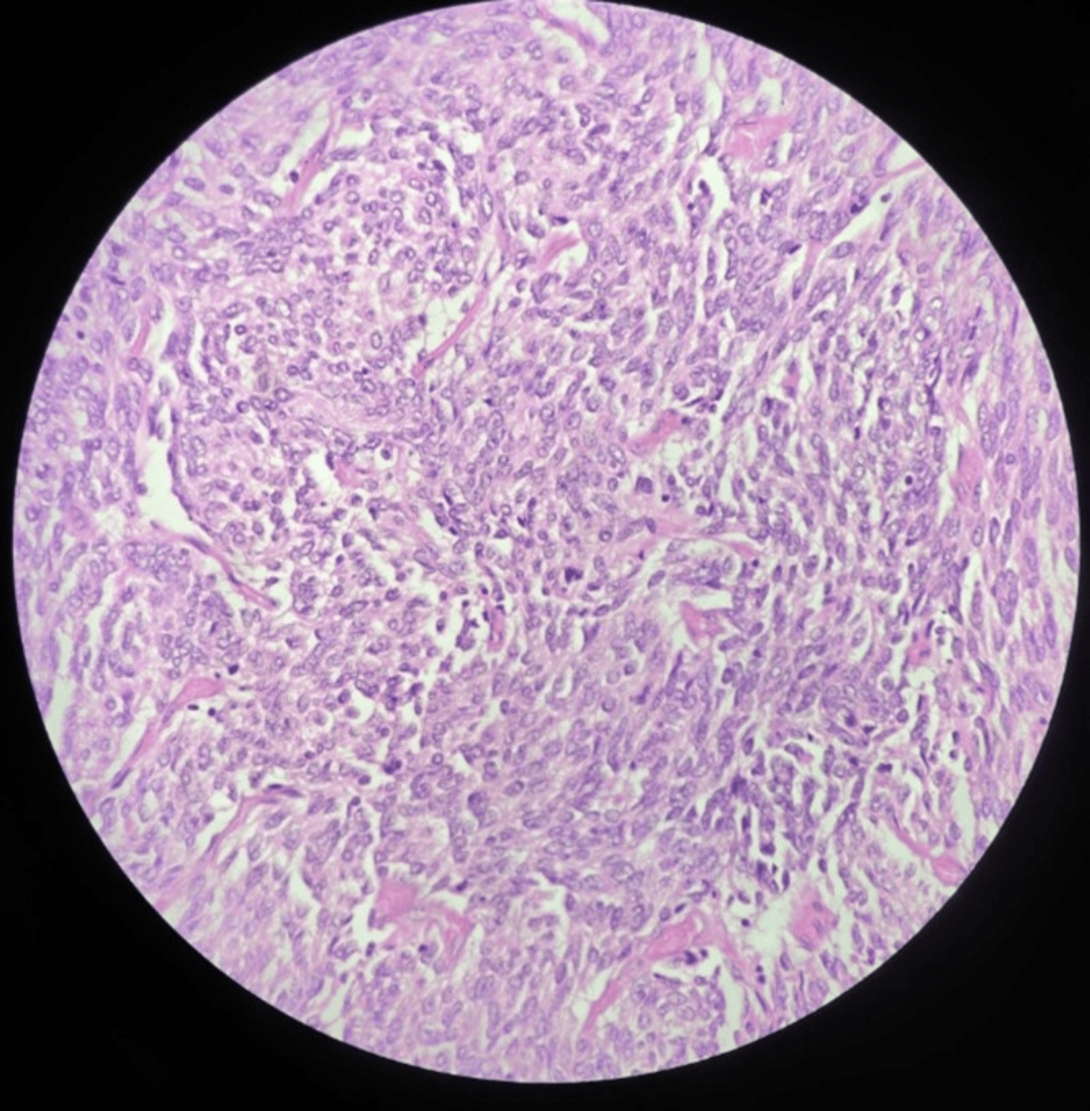
Microscopy (H&E, 40× power) demonstrating tumor cells with eosinophilic cytoplasm and vesicular nuclei H&E: hematoxylin and eosin

Microscopic examination demonstrated a neoplasm composed of spindle to epithelioid cells arranged in fascicles (Figure 9), with moderate eosinophilic cytoplasm and vesicular nuclei (Figure 10). Increased mitotic activity (six mitoses/50 high-power field (HPF)) and areas of necrosis were present. Multifocal tumor deposits showed similar morphology.

**Figure 11:**
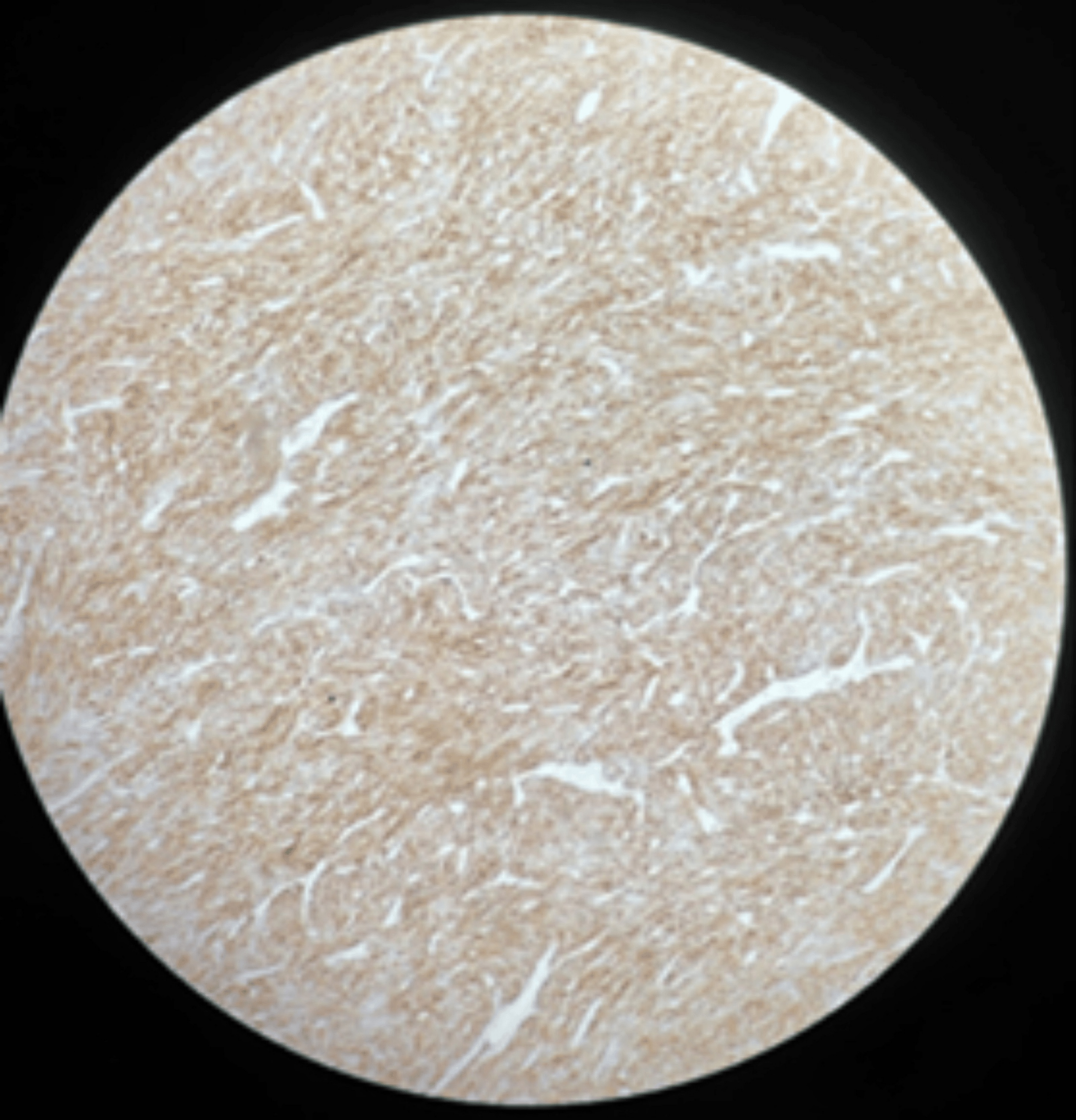
Immunohistochemistry staining showing strong CD117 positivity

**Figure 12:**
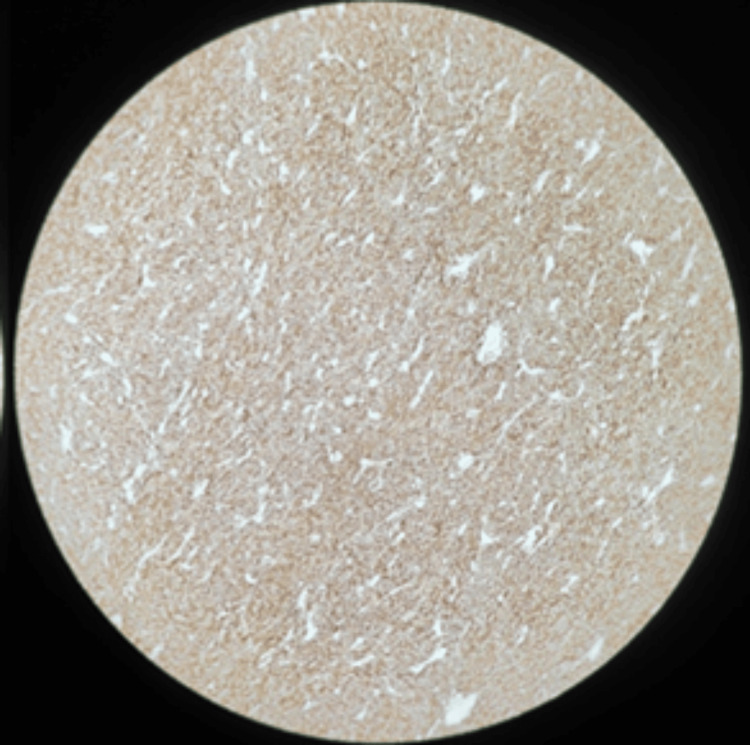
Immunohistochemistry staining showing strong DOG1 positivity

Immunohistochemistry revealed strong positivity for CD117 (Figure 11) and DOG1 (Figure 12), confirming the diagnosis of GIST. The Ki-67 labeling index was elevated at 34%, indicating high proliferative activity. The tumor was classified as high grade (G2) due to mitotic activity of six mitoses/50 HPF, with staging of ypT4(m)N0 in view of prior imatinib therapy. Tumor rupture was confirmed histologically by disruption of the tumor capsule and surface ulceration, and overall features were consistent with a high-risk lesion.

## Discussion

The imaging findings in this case illustrate the characteristic features of complicated GIST. Larger tumors frequently demonstrate heterogeneous enhancement due to necrosis and hemorrhage, reflecting rapid growth outpacing vascular supply [1,3]. The presence of intratumoral gas is an uncommon but critical finding that suggests communication with the bowel lumen or infection within necrotic tumor tissue. When accompanied by pneumoperitoneum, it indicates disruption of tumor integrity and rupture into the peritoneal cavity [3].

Small-bowel GISTs tend to exhibit more aggressive behavior than gastric tumors and are more prone to complications such as bleeding, perforation, and rupture [2]. Tumor rupture results in peritoneal seeding and is associated with a significantly increased risk of recurrence, making it one of the most important prognostic indicators in GIST [5].

In this case, the presence of multiple mesenteric and pelvic lesions, along with peritoneal thickening, is consistent with peritoneal dissemination. The absence of lymphadenopathy aligns with the known metastatic pattern of GIST, which typically involves the peritoneum and liver rather than lymph nodes [1,9].

CT plays a pivotal role in identifying these features, allowing differentiation from other small-bowel neoplasms such as lymphoma (segmental/diffuse wall thickening, homogeneous, aneurysmal bowel dilatation, associated lymphadenopathy); adenocarcinoma (asymmetric wall thickening with or without small bowel obstruction); intra-abdominal abscess (unilocular or multilocular peripherally enhancing fluid collection, internal gas, surrounding fat stranding); and inflammatory bowel perforation (absence of mass lesion, segmental wall thickening with fat stranding and extraluminal air).

Postoperatively, high-risk ruptured GIST warrants tyrosine kinase inhibitor therapy (e.g., imatinib) due to the high risk of recurrence [10]. Long-term follow-up with serial imaging is recommended, as rupture is an independent adverse prognostic factor. Recognition of rupture and dissemination is essential for guiding urgent surgical management and subsequent therapy [8].

## Conclusions

This case demonstrates a rare and aggressive presentation of multifocal ileal GIST with spontaneous rupture, pneumoperitoneum, and peritoneal dissemination. CT findings of intratumoral gas, tumor-bowel communication, and free intraperitoneal air are key indicators of rupture that must alert radiologists in emergency settings. Radiologic-pathologic correlation confirms the aggressive nature of the disease and its high-risk classification. Early recognition of these features is essential for appropriate management and prognostication.
